# Psychosocial and environmental correlates of active and passive transport behaviors in college educated and non-college educated working young adults

**DOI:** 10.1371/journal.pone.0174263

**Published:** 2017-03-20

**Authors:** Dorien Simons, Ilse De Bourdeaudhuij, Peter Clarys, Katrien De Cocker, Bas de Geus, Corneel Vandelanotte, Jelle Van Cauwenberg, Benedicte Deforche

**Affiliations:** 1 Unit Health Promotion and Education, Department of Public Health, Faculty of Medicine and Health Sciences, Ghent University, De Pintelaan 185, Ghent, Belgium; 2 Research unit Physical Activity, Nutrition and Health, Faculty of Physical Education and Physical Therapy, Vrije Universiteit Brussel, Pleinlaan 2, Brussels, Belgium; 3 Research Foundation Flanders, Egmontstraat 5, Brussels, Belgium; 4 Department of Movement and Sport Sciences, Faculty of Medicine and Health Sciences, Ghent University, Watersportlaan 2, Ghent, Belgium; 5 Human Physiology Research Group, Faculty of Physical Education and Physical Therapy, Vrije Universiteit Brussel, Pleinlaan 2, Brussels, Belgium; 6 Central Queensland University, School for Health, Medical and Applied Science, Physical Activity Research Group, Rockhampton QLD, Australia; University of Edinburgh, UNITED KINGDOM

## Abstract

**Background:**

This study aimed to examine potential differences in walking, cycling, public transport and passive transport (car/moped/motorcycle) to work and to other destinations between college and non-college educated working young adults. Secondly, we aimed to investigate which psychosocial and environmental factors are associated with the four transport modes and whether these associations differ between college and non-college educated working young adults.

**Methods:**

In this cross-sectional study, 224 working young adults completed an online questionnaire assessing socio-demographic variables (8 items), psychosocial variables (6 items), environmental variables (10 items) and transport mode (4 types) and duration to work/other destinations. Zero-inflated negative binomial regression models were performed in R.

**Results:**

A trend (p<0.10) indicated that more college educated compared to non-college educated young adults participated in cycling and public transport. However, another trend indicated that cycle time and public transport trips were longer and passive transport trips were shorter in non-college compared to college educated working young adults. In all working young adults, high self-efficacy towards active transport, and high perceived benefits and low perceived barriers towards active and public transport were related to more active and public transport. High social support/norm/modeling towards active, public and passive transport was related to more active, public and passive transport. High neighborhood walkability was related to more walking and less passive transport. Only in non-college educated working young adults, feeling safe from traffic and crime in their neighborhood was related to more active and public transport and less passive transport.

**Conclusions:**

Educational levels should be taken into account when promoting healthy transport behaviors in working young adults. Among non-college educated working young adults, focus should be on increasing active and public transport participation and on increasing neighborhood safety to increase active and public transport use. Among college educated working young adults, more minutes of active transport should be encouraged.

## Background

Insufficient physical activity is one of the leading risk factors for death worldwide, as one in four adults is not meeting the health related physical activity guidelines [1]. In Belgium, 11.4% of all-cause mortality is associated with physical inactivity [2]. Young or emerging adults (ranging from the late teens through the twenties) have shown to be particularly at risk for decreasing physical activity levels as they get older [3, 4]. Young adulthood is a critical period of decision making [5, 6] where major life events such as changes in employment, education, or place of residence can affect physical activity participation [7–10].

Active transport (AT) (i.e. walking, cycling) represents an opportunity to include physical activity into young adults' daily life [11]. AT, including walking or cycling to and from public transport, offers benefits such as higher levels of total moderate-to-vigorous physical activity [11–13], lower odds of being overweight or obese [14, 15], higher levels of cardiovascular fitness [14, 16, 17], an overall reduction in cardiovascular risk [16] and public health benefits due to improved air quality [18, 19]. Despite the many benefits of AT, only 12.2% and 13.6% of young adults (age 18–24) in Flanders (northern half of Belgium) respectively walk and cycle as their main mode of transport. For 34.5% of young adults, driving a car is the main transport mode in everyday life [20]. However, as travel habits are established in young adulthood, it is important to research their transport behavior and encourage sustainable travel choices (AT) that might persist into adulthood and provide long-term benefits [21].

Research on transport habits of young adults is scarce for young adults who are working (as compared to students), presumably because working young adults are not readily accessible through any institutional setting [5]. However, working young adults might be more car dependent because employment often implies the demand to have a driving license (as it is assumed to be associated with adulthood and responsibility) and because of greater financial means and the possibility to own/receive a car [22, 23]. One US study showed that the vast majority (90.4%) of working young adults commute by car [24]. Furthermore, working young adults’ transport behavior might differ according to educational attainment. Level of education reflects achievement of human capital via formal education, accreditation and lived experience [25]. This may influence a person’s health literacy and knowledge about the importance and benefits of AT [26], or the environmental implications of particular transport mode choices. Research found that having completed higher education (college or university), compared to lower education, is associated with more cycling to work in Flemish adults [27], more AT to work in adults from Wales [28] and more AT to all destinations in Australian and UK adults [29, 30]. However, other studies found no or negative relations between educational levels and AT [31–34]. Nevertheless, promoting AT in working young adults, with increased attention for those who are lower educated, seems necessary. To date, the relation between educational level and transport behavior has not yet been studied in working young adults.

To design effective interventions promoting AT in both college and non-college educated working young adults, it is necessary to have a comprehensive understanding of the correlates of AT and of other modes of transport (e.g. public transport) in both target groups [35]. Ecological models state that transport choices are influenced by various factors at multiple levels, including psychosocial and environmental factors [36–38]. Previous studies investigating correlates of transport habits in young adults have focused on studying instead of working young adults [11, 39–41]. One qualitative study did include working young adults [42], but without taking educational level into account. Few AT studies (focusing on adults in general) have investigated moderators such as educational level and in those studies that did, mixed results were found (positive, negative and null associations) [27, 43–46].

Therefore, the aims of this study were (1) to examine differences in walking, cycling, public transport use and passive transport use to work and to other destinations between college and non-college educated working young adults, (2) to investigate which psychosocial and environmental factors are associated with the four transport modes and (3) to investigate whether these associations differ between college and non-college educated working young adults.

## Methods

### Participants

Working young adults (eligible age range: 18–26 years) were recruited from companies across Flanders (Belgium). A list of randomly selected companies that potentially employ many young people (both college and non-college educated or either one or the other) was made based on an internet search (n = 41). Contact was made via e-mail and phone with HR-managers of these companies. After agreement to participate, HR-managers were asked to forward an email with a link to an online questionnaire to the working young adults. Because of the low response rate of companies (n = 13, 30%) and employees, working young adults were also recruited face-to-face in other settings. Researchers went to random commercial and retail businesses to recruit young adult employees. In addition to these recruitment methods, social media (i.e. Facebook) was used in order to increase the sample size. An advertisement banner with an invitation to fill in the online questionnaire was placed on Facebook. Within young adults, social network sites are very popular and therefore an effective way to reach young people and send out online questionnaires [47]. Incentives (three folding bicycles) were raffled among participants who completed the questionnaire. The study protocol was approved by the ethics committee of the university hospital of the Vrije Universiteit Brussel (B.U.N. 143201112745).

### Research procedure and measures

A cross-sectional design was used to collect self-reported data using an online survey (as also described previously in a study among older adolescents [48]). The survey assessed socio-demographic variables, general transport data, transport to work and to other destinations, psychosocial variables and environmental variables (see details below). The questions were based on validated questionnaires [27, 49, 50], but were adjusted to better fit the target group. These adjustments were made according to the results of a prior exploratory qualitative study using focus groups [42].

#### Socio-demographic variables

Self-reported socio-demographic variables included gender, age and nationality (Belgian, other). Educational level was dichotomized into non-college educated (elementary school or secondary school as highest degree) and college educated (college or university as highest degree). Furthermore, living situation was dichotomized into living with (grand)parents and not living with (grand)parents (with partner/alone/other). Living environment was dichotomized into rural area (countryside/village) and urban area (suburban area/city). Participants also reported their height and weight, which was used to calculate Body Mass Index (BMI).

#### General transport data

General transport data included possession of a driving license for a car (yes/no); ownership of moped, car/motorcycle and bicycle (yes/no); and pass ownership for public transport (yes/no) and for bicycle sharing schemes (yes/no).

#### Transport to work and to other destinations

Questions based on the International Physical Activity Questionnaire (IPAQ, long version), which has been validated in Flemish adults [51], were used to assess transport behavior to work and to destinations other than work. Frequency (days/week) and duration (minutes/day) of walking, cycling, public transport and passive transport (all questioned separately) within the last seven days were assessed. If participants combined transport modes (e.g. combining public transport and AT), they were asked to report each component separately. All information on transport modes was subdivided in four main groups: walking, cycling, public transport (train/bus/tram/subway) and passive transport (car/motorcycle/moped).

#### Psychosocial variables

The following psychosocial variables in relation to active and passive transport to all destinations were assessed: self-efficacy, social norm, modeling, social support, perceived benefits and perceived barriers. They were selected based on the Attitude-Social influence-self-Efficacy (ASE) model [52]. A summary of the measures of psychosocial variables and Cronbach’s alpha for internal consistency are shown in Table 1. Averages of item scores were used for the present data analyses. Self-efficacy was assessed by asking participants how confident they were to choose AT over other transport modes in 11 potentially difficult situations (i.e. bad weather, darkness, when tired). Social norm was measured by asking if participants believed that partner/parents/siblings/friends/colleagues (asked separately) expect them to (a) walk or cycle; (b) take a car/motorcycle/moped; (c) use public transport. Modeling was assessed by asking how frequently partner/parents/siblings/friends/colleagues (asked separately) (a) walk or cycle; (b) take a car/motorcycle/moped; (c) use public transport. To investigate social support, participants were asked (1) how often partner/parents/siblings/friends/colleagues (asked separately) encourage them to (a) walk or cycle; (b) take a car/motorcycle/moped; (c) use public transport and (2) how often they do this together with them. To measure perceived benefits, participants were asked about potential benefits (i.e. health, cost, independence) of (a) walking or cycling; (b) taking the car/motorcycle/moped; (c) using public transport. Perceived barriers were assessed by asking participants about potential barriers (i.e. time, accidents, delays) of (a) walking or cycling; (b) taking the car/motorcycle/moped; (c) using public transport.

**Table 1:** Summary of psychosocial and environmental measures, Cronbach’s alpha (*α)* and mean scores (SD).

Scale (composition)		Response category	α	M (SD)
**Psychosocial**				
Self-efficacy				
*active transport*	11 items	five-point scale from 1 (know I cannot do it) to 5 (know I can do it)	0.89	3.45 (0.92)
Social norm				
*active transport*	5 items	five-point scale from 1 (strongly disagree) to 5 (strongly agree)	0.90	2.78 (1.14)
*public transport*	5 items	five-point scale from 1 (strongly disagree) to 5 (strongly agree)	0.93	2.35 (1.12)
*passive transport*	5 items	five-point scale from 1 (strongly disagree) to 5 (strongly agree)	0.96	2.39 (1.18)
Social modeling				
*active transport*	5 items	five-point scale: never or once per year, 1 time per month, several times per month, several times per week, almost every day	0.74	2.98 (0.99)
*public transport*	5 items	five-point scale: never or once per year, 1 time per month, several times per month, several times per week, almost every day	0.63	2.45 (0.92)
*passive transport*	5 items	five-point scale: never or once per year, 1 time per month, several times per month, several times per week, almost every day	0.86	4.06 (1.02)
Social support				
*active transport*	5 items	five-point scale from 1 (never) to 5 (always)	0.79	2.21 (0.69)
*public transport*	5 items	five-point scale from 1 (never) to 5 (always)	0.86	1.84 (0.70)
*passive transport*	5 items	five-point scale from 1 (never) to 5 (always)	0.89	2.48 (0.76)
Perceived benefits				
*active transport*	18 items	five-point scale from 1 (strongly disagree) to 5 (strongly agree)	0.88	3.68 (0.66)
*public transport*	6 items	five-point scale from 1 (strongly disagree) to 5 (strongly agree)	0.76	2.61 (0.82)
*passive transport*	7 items	five-point scale from 1 (strongly disagree) to 5 (strongly agree)	0.83	3.50 (0.81)
Perceived barriers				
*active transport*	22 items	five-point scale from 1 (never) to 5 (always)	0.90	2.17 (0.61)
*public transport*	8 items	five-point scale from 1 (never) to 5 (always)	0.83	2.91 (0.80)
*passive transport*	11 items	five-point scale from 1 (never) to 5 (always)	0.85	2.30 (0.76)
**Environmental**				
Residential density	3 items	five-point scale from 1 (no houses/ apartments) to 5 (all houses/apartments)		2.33 (0.86)
Land use mix diversity	8 items	five-point scale: 1–5 min, 6–10 min, 11–20 min, 20–30 min, > 30 minutes		3.49 (0.93)
Land use mix access	6 items	four-point scale: strongly disagree, somewhat disagree, somewhat agree, strongly agree		3.04 (0.58)
Street connectivity	5 items	four-point scale: strongly disagree, somewhat disagree, somewhat agree, strongly agree		2.73 (0.43)
Walking and cycling facilities	12 items	four-point scale: strongly disagree, somewhat disagree, somewhat agree, strongly agree		2.47 (0.40)
Aesthetics	4 items	four-point scale: strongly disagree, somewhat disagree, somewhat agree, strongly agree		2.70 (0.55)
Perceived safety from traffic	8 items	four-point scale: strongly disagree, somewhat disagree, somewhat agree, strongly agree		2.64 (0.46)
Perceived safety from crime	6 items	four-point scale: strongly disagree, somewhat disagree, somewhat agree, strongly agree		3.28 (0.62)
Facilities at work	8 items	two-point scale: 1 (yes) and 2 (no)		0.52 (0.28)

α = Cronbach’s alpha for internal consistency

M (SD) = Mean (Standard Deviation)

#### Perceived environmental variables

A summary of the measures of environmental variables are shown in Table 1. Perceived environmental variables were assessed using questions derived from validated questionnaires: the European environmental questionnaire (ALPHA questionnaire) [53] and the Neighborhood Environment Walkability Scale (NEWS, original version) [54, 55]. ‘Neighborhood’ was defined as ‘the environment within a walking or cycling distance of 10–15 minutes from home’. Data were cleaned and analyzed according to the ALPHA environmental questionnaire Manual [56] and the NEWS scoring procedures [57]. The following perceived environmental variables were assessed: residential density (ALPHA), land use mix diversity (ALPHA), land use mix access (NEWS), street connectivity (NEWS), walking and cycling facilities (ALPHA and NEWS), aesthetics (ALPHA), safety from traffic (NEWS) (a high perceived safety from traffic = feeling safe from problems such as speed of traffic in neighborhood) and safety from crime (NEWS) (a high perceived safety from crime = feeling safe from problems such as crime prevalence in the neighborhood). Furthermore, facilities at work (e.g., showers, bicycle storage, car park) and self-reported distance to work (in kilometers) were assessed (ALPHA).

### Data analysis

To examine differences in transport behavior between college and non-college educated working young adults, and to investigate the associations of psychosocial and environmental factors with walking, cycling, public transport and passive transport, zero-inflated negative binomial (ZINB) regression models were used. Analyses were done using R with the package ‘pscl’ [58]. ZINB models were used as the dependent variables were positively skewed and contained a large number of zero values. Vuong tests supported the need to use zero-inflated regression models [59] and Akaike’s Information Criterium (AIC) showed that a ZINB model was preferred over a zero-inflated poisson model. ZINB models evaluate the relationships of the independent variables (psychosocial and environmental factors) with the odds of non-participation in walking, cycling, public transport use and passive transport use to work and to other destinations. Simultaneously, ZINB models estimate the relationships of the independent variables with minutes per week participated in these transport modes for those who did make use of these transport modes. The zero-inflated model and the negative binomial model might differ in predictors. Hence, one ZINB model might yield two regression coefficients for the independent variables: an odds ratio (OR) (for the relationship between the independent variable and the odds of not participating in walking, cycling, using passive transport or using public transport) and a negative-binomial model regression coefficient (exponentiated beta coefficient representing the proportional change in minutes/week walking, cycling, using public transport or using passive transport with a one-unit increase in the independent variable for those who did participate in these transport modes).

All analyses were conducted separately for the four dependent variables (walking, cycling, public transport and passive transport) and separately for trips to work and to other destinations. Initially, a model was developed for each dependent variable with all demographic variables (gender, age, BMI, living situation, living environment, car ownership, bicycle sharing schemes pass ownership, public transport pass ownership). In all of the following models, demographic variables that were significantly related to the outcome in the initial step were included as covariates. To answer the first study aim (exploring differences in transport behavior between college and non-college educated working young adults), a model was estimated that included educational level and relevant covariates. To investigate the second and third aim (investigating psychosocial and environmental correlates and differences between college and non-college educated working young adults), two basic models, one including all psychosocial variables plus educational level and one including all environmental variables plus educational level, were developed for each dependent variable (16 models). Next, the interaction term between educational level and each of the independent variables were added separately to the two basic models. Following that, all significant interaction terms observed in the previous step were entered simultaneously into one of the two basic models. Finally, all significant independent variables and interaction terms of the two basic models were entered simultaneously into a final model. The results of these 8 final models (one for each dependent variable) were presented. Distance to work and facilities at work were only included in the models for transport to work. Level of significance was set at p<0.05. A trend for significance was considered at p<0.10.

## Results

In total 355 working young adults participated in the survey of which 224 (63%) completed the entire questionnaire. Table 2 presents socio-demographic characteristics, general transport data and data on transport to work and other destinations for the whole sample and separately for college and non-college educated working young adults. In total, 56% of the sample was female with a mean age of 25 years, 39% lived with their (grand)parents and 50% lived in a rural area.

**Table 2:** Descriptive characteristics of the sample (%, Mean (SD)).

	All	College educated	Non-college educated
	n = 224	n = 153 (68%)	n = 71 (32%)
**Socio-demographic characteristics**			
Gender (% female)	55.8	59.5	47.9
Age (years)	24.6 (1.4)	24.8 (1.1)	24.4 (1.9)
BMI (kg/m^2^)	22.9 (3.3)	22.6 (3.2)*	23.5 (3.4)*
Nationality (% Belgian)	96.9	95.4	100.0
Living situation (% living with their (grand)parents)	39.3	37.9	42.3
Living environment (% living in rural area)	49.6	47.7	53.5
**Transport in general**			
*Car/motorcycle*			
Driving license (%)	84.8	86.9	80.3
Ownership (%)	64.3	64.1	64.8
*Moped*			
Driving license (%)	23.2	20.3	29.6
Ownership (%)	3.6	3.3	4.2
*Bicycle*			
Ownership (%)	93.8	96.1	88.7
Ownership public transport pass (%)	37.5	36.6	39.4
Ownership bicycle sharing schemes pass (%)	8.5	10.5	4.2
Kilometres to work	21.1 (48.6)	18.9 (17.8)	25.8 (82.0)
**Transport to work (mode and duration)**			
Participants who walked (n)	39	29	10
Amount walking (minutes/week)	90.8 (90.1)	82.6 (66.5)	114.5 (140.2)
Participants who cycled (n)	78	60*	18*
Amount cycling (minutes/week)	135.3 (108.0)	31.1 (102.5)	149.2 (126.9)
Participants who made use of public transport (n)	67	46	21
Amount public transport use (minutes/week)	332.1 (235.3)	307.6 (247.0)	385.7 (202.7)
Participants who made use of passive transport (n)	131	87	44
Amount passive transport use (minutes/week)	232.6 (169.2)	253.0 (174.1)°	192.9 (153.8)°
**Transport to other destinations (mode and duration)**			
Participants who walked (n)	117	87	40
Amount walking (minutes/week)	106.9 (168.8)	96.0 (142.6)	130.8 (215.4)
Participants who cycled (n)	92	66	26
Amount cycling (minutes/week)	117.9 (130.0)	96.8 (91.6)°	171.7 (188.4)°
Participants who made use of public transport (n)	82	60	22
Amount public transport use (minutes/week)	196.5 (221.4)	171.8 (214.3)°	263.9 (231.4)°
Participants who made use of passive transport (n)	187	128	60
Amount passive transport use (minutes/week)	211.6 (267.9)	222.7 (281.5)	187.8 (236.4)

° p<0.10

* p<0.05

### Differences in choice of transport mode between college and non-college educated working young adults (Table 3)

The logit model showed a trend that, for cycling to work and for public transport to other destinations, being higher educated was associated with 47% lower odds of being a non-participant. In other words, college educated working young adults were more likely to cycle to work and more likely to use public transport to other destinations than non-college educated participants. Among those who did walk, cycle, use public transport or use passive transport, it was found that college educated working young adults cycled 44% less minutes/week to other destinations, used public transport 29% less minutes/week to work (trend) and commuted 38% more minutes/week passively than non-college educated working young adults.

**Table 3:** Differences in mode and duration of transport mode between non-college educated (reference category) and college educated working young adults.

	To work		To other destinations	
Outcomes	Logit model: OR of being non-participant^a^^,^ ^c^ (95% CI)	Negative binomial model: minutes/week^b^^,^ ^c^ (95% CI)	Logit model: OR of being non-participant^a^^,^ ^c^ (95% CI)	Negative binomial model: minutes/week^b^^,^ ^c^ (95% CI)
walking	0.53 (0.22, 1.27)	0.67 (0.37, 1.21)	1.09 (0.60, 1.98)	0.73 (0.49, 1.08)
cycling	0.53 (0.26, 1.08)°	0.81 (0.55, 1.20)	0.73 (0.38, 1.41)	0.56 (0.38, 0.85)**
public transport	0.82 (0.36, 1.86)	0.71 (0.49, 1.03)°	0.53 (0.25, 1.12)°	0.85 (0.52, 1.39)
passive transport	1.39 (0.67, 2.90)	1.38 (1.05, 1.81)*	0.94 (0.41, 2.16)	1.23 (0.92, 1.65)

OR = odds ratio

CI = confidence interval

° p<0.10

* p<0.05

** p<0.01.

^a^ Logit model: ZINB models evaluate the correlates of the odds of non-participation in using a specific transport mode to work or to other destinations.

^b^ Negative binomial model: simultaneously, among participants who did use that transport mode to work or to other destinations, ZINB models evaluate the correlates of weekly minutes using that transport mode to work or to other destinations. Exponentiated negative binomial model parameters represent the proportional increase in minutes/week transport mode use to work or to other destinations with a one-unit increase in the predictor. The model for commuting was adjusted for distance to work.

^c^ Estimate represents the relationships between educational level (with non-college educated as the reference category) and the outcomes.

### Correlates of walking

#### Main effects of psychosocial and environmental correlates on walking

Table 4 shows that for walking to work, a one-unit increase in perceived safety from traffic was associated with 73% more minutes/week walking among those who did walk.

**Table 4:** Associations of psychosocial and environmental variables and the interactions terms with walking.

	Walking to work		Walking to other destinations	
	Logit model: OR of being non-participant in walking^a^ (95% CI)	Negative binomial model: minutes/week walking^b^ (95% CI)	Logit model: OR of being non-participant in walking^a^ (95% CI)	Negative binomial model: minutes/week walking^b^ (95% CI)
**Psychosocial**				
Self-efficacy			0.57 (0.39, 0.83)**	
social support				1.41 (1.06, 1.88)*
social norm	0.79 (0.54, 1.16)			
Modeling	0.65 (0.41, 1.03)		0.79 (0.57, 1.11)	
perceived benefits		3.33 (1.13, 9.79)*, ^c^		
Perceived barriers				0.54 (0.32, 0.89)*, ^c^
**Evironmental**				
land use mix diversity			0.62 (0.43, 0.90)*	0.77 (0.62, 0.95)*
street connectivity	0.45 (0.17, 1.17)			
walking and cycling facilities		0.75 (0.46, 1.20)	3.09 (1.34, 7.13)**	
Aesthetics				0.69 (0.49, 0.96)*
safety from traffic		1.73 (1.21, 2.49)**		
facilities at work		0.96 (0.37, 2.49)		
Distance	0.99 (0.98, 1.00)	0.99 (0.99, 1.00)		
**Interaction terms**				
perceived benefits*educational level		0.23 (0.07, 0.80)*		
perceived barriers*educational level				2.41 (1.30, 4.47)**

OR = odds ratio

CI = confidence interval

* p<0.05

** p<0.01.

^a^ Logit model: ZINB models evaluate the correlates of the odds of non-participation in walking to work or to other destinations.

^b^ Negative binomial model: simultaneously, among participants who did walk to work or to other destinations, ZINB models evaluate the correlates of weekly minutes walking to work or to other destinations. Negative binomial model parameters represent the proportional increase in minutes/week walking to work or to other destinations with a one-unit increase in the predictor. The model for commuting was adjusted for distance to work.

^c^ Reference category is non-college educated

For walking to other destinations, working young adults with a higher self-efficacy towards AT, those perceiving higher land use mix diversity and those perceiving less walking and cycling facilities were more likely to walk. Among those who did walk, a one-unit increase in social support was associated with 41% more minutes/week walking. In addition, a one-unit increase in land use mix diversity and a one-unit increase in aesthetics was associated with respectively 23% and 31% less minutes/week walking.

#### Interaction effects with educational level

For walking to work, no interaction effects between correlates and educational level were found in the logit model. Among those who did walk to work, an interaction effect between educational level and perceived benefits regarding AT was found. In non-college educated working young adults, a one-unit increase in perceived benefits regarding AT was associated with 233% more minutes/week walking. There was no effect of perceived benefits in college educated working young adults (95% CI = 0.51, 1.17).

For walking to other destinations, no interaction effects between correlates and educational level were found in the logit model. Among those who did walk to other destinations, an interaction effect was found between educational level and perceived barriers regarding AT. In non-college educated working young adults, a one-unit increase in perceived barriers regarding AT was associated with 46% less minutes/week walking. There was no effect of perceived barriers in college educated participants (95% CI = 0.92, 1.84).

### Correlates of cycling

#### Main effects of psychosocial and environmental correlates on cycling

Table 5 shows that for cycling to work, working young adults with a higher self-efficacy towards AT, those perceiving more facilities at work in favor of walking and cycling and those living closer to work were more likely to cycle. Among those who did cycle to work, a one-unit increase in perceived benefits regarding AT and in walking and cycling facilities was associated with respectively 52% and 70% more minutes/week cycling.

**Table 5:** Associations of psychosocial and environmental variables and the interaction terms with cycling.

	Cycling to work		Cycling to other destinations	
	Logit model: OR of being non-participant in cycling^a^ (95% CI)	Negative binomial model: minutes/week cycling^b^ (95% CI)	Logit model: OR of being non-participant in cycling^a^ (95% CI)	Negative binomial model: minutes/week cycling^b^ (95% CI)
**Psychosocial**				
Self-efficacy	0.29 (0.15, 0.56)***		0.52 (0.29, 0.96)*	
social norm				1.50 (1.02, 2.22)*, ^c^
perceived benefits		1.52 (1.08, 2.16)*	2.28 (0.61, 8.58)^c^	1.49 (1.08, 2.05)*
Perceived barriers	2.13 (0.98, 4.63)		3.41 (1.58, 7.38)**	0.72 (0.52, 0.99)*
**Evironmental**				
land use mix diversity			1.74 (1.00, 3.04)*	
land use mix access			0.44 (0.18, 1.06)	
walking and cycling facilities		1.70 (1.05, 2.76)*	0.38 (0.12, 1.18)	
safety from traffic		0.79 (0.52, 1.22)	0.15 (0.03, 0.68)*, ^c^	0.64 (0.44, 0.95)*
facilities at work	0.06 (0.01, 0.30)***	1.72 (0.86, 3.45)		
Distance	1.03 (1.01, 1.06)**	1.00 (0.98, 1.01)		
**Interaction terms**				
social norm*educational level				0.67 (0.43, 1.03°
perceived benefits*educational level			0.19 (0.04, 0.83)*	
safety from traffic*educational level			5.97 (0.96, 37.20°	

OR = odds ratio

CI = confidence interval

* p<0.05

** p<0.01

*** p<0.001.

^a^ Logit model: ZINB models evaluate the correlates of the odds of non-participation in cycling to work or to other destinations.

^b^ Negative binomial model: simultaneously, among participants who did cycle to work or to other destinations, ZINB models evaluate the correlates of weekly minutes cycling to work or to other destinations. Negative binomial model parameters represent the proportional increase in minutes/week cycling to work or to other destinations with a one-unit increase in the predictor. The model for commuting was adjusted for distance to work.

^c^ Reference category is non-college educated

For cycling to other destinations, those with a higher self-efficacy towards AT, those perceiving less barriers towards AT and those perceiving a lower land use mix diversity were more likely to cycle. Among those who did cycle to other destinations, a one-unit increase in perceived benefits regarding AT was associated with 49% more minutes/week cycling. Additionally, a one-unit increase in perceived barriers regarding AT and in safety from traffic was associated with respectively 28% and 36% less minutes/week cycling to other destinations.

#### Interaction effects with educational level

For cycling to work, no interaction effects were found with educational level. For cycling to other destinations, an interaction effect was found between educational level and perceived benefits and a trend towards an interaction effect was found between educational level and safety from traffic. In non-college educated working young adults, there was no effect of perceived benefits, but those perceiving a higher safety from traffic were more likely to cycle. In college educated working young adults, those perceiving more benefits regarding AT were more likely to cycle (56% lower odds, 95% CI = 0.19, 0.98), but no effect of safety from traffic was found (95% CI = 0.30, 2.74). Among those who did cycle to other destinations, a trend towards an interaction effect was found between educational level and social norm. In non-college educated working young adults, a one-unit increase in social norm regarding AT was associated with 50% more minutes/week cycling, but there was no effect of social norm in college educated participants (95% CI = 0.84, 1.19).

### Correlates of public transport use

#### Main effects of psychosocial and environmental correlates on public transport use

Table 6 shows that for public transport use to work, working young adults perceiving a higher social norm were more likely to use public transport. Among those who did commute by public transport, a one-unit increase in residential density and a 1 km increase in distance to work was associated with respectively 23% and 2% more minutes/week public transport use. In addition, a one-unit increase in facilities at work in favor of walking and cycling was associated with 49% less minutes/week public transport use.

**Table 6:** Associations of psychosocial and environmental variables and the interaction terms with public transport.

	Public transport to work		Public transport to other destinations	
	Logit model: OR of being non-participant in public transport^a^ (95% CI)	Negative binomial model: minutes/week public transport use^b^ (95% CI)	Logit model: OR of being non-participant in public transport^a^ (95% CI)	Negative binomial model: minutes/week public transport use^b^ (95% CI)
**Psychosocial**				
social support			0.39 (0.21, 0.73)**	
social norm	0.51 (0.33, 0.81)**			1.42 (1.17, 1.73)***
Modeling	1.13 (0.55, 2.33)^c^		0.59 (0.35, 0.99)*	
perceived benefits	0.49 (0.17, 1.37)^c^	0.88 (0.69, 1.12)		0.54 (0.40, 0.71)***
perceived barriers				1.42 (1.06, 1.90)*
**Evironmental**				
residential density		1.23 (1.03, 1.48)*	0.64 (0.37, 1.13)	1.18 (0.94, 1.48)
land use mix diversity		1.26 (0.87, 1.81)^c^	5.35 (1.99, 14.38)***, ^c^	
land use mix access			0.52 (0.22, 1.24)	
street connectivity				1.43 (0.93, 2.19)
walking and cycling facilities			1.79 (0.64, 4.99)	
safety from crime		1.76 (1.08, 2.88)*, ^c^	0.23 (0.06, 0.90)*, ^c^	
facilities at work		0.51 (0.27, 0.97)*		
Distance	1.00 (0.98, 1.02)^c^	1.02 (1.01, 1.03)***		
**Interaction terms**				
modeling*educational level	0.32 (0.11, 0.89)*			
perceived benefits*educational level	2.85 (0.82, 9.84°			
land use mix diversity*educational level		0.68 (0.43, 1.07°	0.24 (0.09, 0.64)**	
safety from crime*educational level		0.53 (0.30, 0.94)*	4.77 (1.01, 22.59)*	
distance*educational level	0.96 (0.92, 0.99)*			

OR = odds ratio

CI = confidence interval

* p<0.05

** p<0.01

*** p<0.001.

^a^ Logit model: ZINB models evaluate the correlates of the odds of non-participation in public transport to work or to other destinations.

^b^ Negative binomial model: simultaneously, among participants who did use public transport to work or to other destinations, ZINB models evaluate the correlates of weekly minutes public transport use to work or to other destinations. Negative binomial model parameters represent the proportional increase in minutes/week public transport use to work or to other destinations with a one-unit increase in the predictor. The model for commuting was adjusted for distance to work.

^c^ Reference category is non-college educated

For public transport to other destinations, working young adults perceiving more social support and more modeling were more likely to use public transport. Among those who did use public transport to other destinations, a one-unit increase in social norm and in perceived barriers was associated with 42% more minutes/week public transport use. Furthermore, a one-unit increase in perceived benefits was associated with 46% less minutes/week public transport use.

#### Interaction effects with educational level

For public transport use to work, interaction effects of modeling and distance to work with educational level were found. No effects of modeling and distance were found in non-college educated participants, but college educated working young adults perceiving more modeling for public transport and living further away from work (respectively 64% (95% CI = 0.18, 0.72) and 4% (95% CI = 0.93, 0.98) lower odds) were more likely to use public transport. Although a trend towards an interaction effect was found between educational level and perceived benefits, no effect of perceived benefits was found in non-college or college (95% CI = 0.72, 2.66) educated participants. Among those who did commute by public transport, an interaction effect was found between educational level and safety from crime. In non-college educated working young adults, a one-unit increase in safety from crime was associated with 76% more minutes/week public transport use, but there was no effect of safety from crime in college educated participants (95% CI = 0.68, 1.29). Although a trend was found towards an interaction effect between educational level and land use mix diversity, there was no effect of land use mix diversity in non-college or college (95% CI = 0.64, 1.14) educated working young adults.

For public transport use to other destinations, interaction effects of land use mix diversity and safety from crime with educational level were found. Non-college educated working young adults perceiving a lower land use mix diversity and a higher safety from crime were more likely to use public transport. No effects of land use mix diversity (95% CI = 0.68, 2.45) and safety from crime (95% CI = 0.48, 2.49) were found in college educated participants. Among those who did use public transport to other destinations, no interaction effects were found with educational level.

### Correlates of passive transport

#### Main effects of psychosocial and environmental correlates on passive transport

Table 7 shows that for passive transport to work, working young adults perceiving more modeling for passive transport, living in a less densely built neighborhood, perceiving a lower land use mix access and a lower safety from crime were more likely to use passive transport. Among those who did commute passively, a one-unit increase in social norm regarding passive transport was associated with 18% more minutes/week using passive transport. In addition, a one-unit increase in land use mix diversity and in facilities at work in favor of walking and cycling was associated with respectively 17% and 36% less minutes/week using passive transport.

**Table 7:** Associations of psychosocial and environmental variables and the interaction terms with passive transport.

	Passive transport to work		Passive transport to other destinations	
	Logit model: OR of being non-participant in passive transport^a^ (95% CI)	Negative binomial model: minutes/week passive transport use^b^ (95% CI)	Logit model: OR of being non-participant in passive transport^a^ (95% CI)	Negative binomial model: minutes/week passive transport use^b^ (95% CI)
**Psychosocial**				
social norm	2.13 (1.08, 4.23)*, ^c^	1.18 (1.09, 1.28)***	0.63 (0.42, 0.96)*	1.21 (1.09, 1.34)***
Modeling	0.66 (0.44, 0.99)*			
perceived benefits	0.60 (0.33, 1.08)		0.43 (0.23, 0.79)**	
perceived barriers			0.59 (0.31, 1.14)	0.74 (0.56, 0.99)*, ^c^
**Evironmental**				
residential density	1.78 (1.02, 3.12)*			
land use mix diversity		0.83 (0.74, 0.94)**		
land use mix access	3.06 (1.29, 7.24)*			0.92 (0.71, 1.19)
safety from traffic	6.30 (1.19, 33.31)*, ^c^		5.26 (1.01, 27.54)*, ^c^	
safety from crime	4.15 (1.82, 9.45)***	1.35 (1.01, 1.80)*, ^c^		0.72 (0.58, 0.90)**
facilities at work	2.98 (0.62, 14.41)	0.64 (0.43, 0.95)*		
Distance	0.98 (0.95, 1.00)	1.00 (0.99, 1.00)^c^		
**Interaction terms**				
social norm*educational level	0.30 (0.13, 0.69)**			
perceived barriers*educational level				1.66 (1.17, 2.35)**
safety from traffic*educational level	0.02 (0.00, 0.16)***		0.08 (0.01, 0.60)*	
safety from crime*educational level		0.59 (0.42, 0.83)**		
distance*educational level		1.02 (1.02, 1.03)***		

OR = odds ratio

CI = confidence interval

* p<0.05

** p<0.01

*** p<0.001.

^a^ Logit model: ZINB models evaluate the correlates of the odds of non-participation in passive transport to work or to other destinations.

^b^ Negative binomial model: simultaneously, among participants who did use passive transport to work or to other destinations, ZINB models evaluate the correlates of weekly minutes passive transport use to work or to other destinations. Negative binomial model parameters represent the proportional increase in minutes/week passive transport use to work or to other destinations with a one-unit increase in the predictor. The model for commuting was adjusted for distance to work.

^c^ Reference category is non-college educated

For passive transport to other destinations, working young adults perceiving a higher social norm and more benefits regarding passive transport were more likely to travel passively. Among those who did use passive transport to other destinations, a one-unit increase in social norm and safety from crime was associated with respectively 21% more minutes/week and 28% less minutes/week using passive transport.

#### Interaction effects with educational level

For passive transport to work, interaction effects of social norm and safety from traffic with educational level were found. Non-college educated working young adults perceiving a lower social norm towards passive transport were more likely to commute passively. There was no effect of social norm in college educated participants (95% CI = 0.39, 1.05). In non-college educated working young adults, those perceiving a lower safety from traffic were more likely to use passive transport, but in college educated participants, the opposite effect was found (95% CI = 0.03, 0.44). Among those who did use passive transport to work, interaction effects of safety from crime and distance to work with educational level were found. In non-college educated working young adults, a one-unit increase in safety from crime was associated with 35% more minutes/week using passive transport, whereas in college educated participants, this was associated with 20% less minutes/week using passive transport (95% CI = 0.65, 0.97). No effect of distance was found in non-college educated working young adults, but in college educated participants, a one kilometer increase in distance to work was associated with 2% more minutes/week using passive transport to work.

For passive transport to other destinations, an interaction effect was found between educational level and safety from traffic. Non-college educated working young adults perceiving a lower safety from traffic were more likely to use passive transport, but there was no effect of safety from traffic in college educated participants (95% CI = 0.13, 1.35). Among those who did use passive transport to other destinations, an interaction effect was found between educational level and perceived barriers. In non-college educated working young adults, a one-unit increase in perceived barriers was associated with 26% less minutes/week using passive transport. No effect of perceived barriers was found in college educated participants (95% CI = 0.99, 1.54).

## Discussion

First, we examined differences in walking, cycling, public transport and passive transport to work and to other destinations between college and non-college educated working young adults. A trend (p<0.10) showed that college educated compared to non-college educated participants were more likely to cycle to work (39.2% vs. 25.4%) and to use public transport to other destinations (39.2% vs. 31.0%). Public transport use can, in combination with AT, contribute to a sustainable and healthy transport behavior. The additional minutes of walking or cycling before and after public transport trips may help to increase physical activity levels and reduce health risks [60–62]. Our results also showed that, among those who did use the corresponding transport mode, non-college educated participants cycled longer to other destinations and made longer public transport trips (trend) and shorter passive transport trips to work than their college educated counterparts. Making longer AT trips (and shorter passive transport trips) may result in greater health effects [17], but it only applies to those actually participating in AT. Thus, although it is good that non-college educated participants make longer AT trips, it is disadvantageous that fewer of them do it. Therefore, future interventions targeting a healthy transport behavior should firstly focus on the adoption of a habit of overall participation in AT and in public transport among non-college educated working young adults. To do that, it might be necessary to increase their knowledge on the importance and benefits of these sustainable transport mode [26]. In higher educated working young adults, longer AT trips, although still within a feasible walking or cycling distance [63], could be encouraged when possible (also to destinations other than work).

Overall, both psychosocial and environmental variables were related to the four transport modes in college and non-college educated working young adults. However, results suggest that psychosocial variables are more important than environmental variables for transport to destinations other than work. For transport to work, environmental variables, especially those related to the workplace (distance to work, facilities at work) seemed more important than psychosocial variables. Travelling to other destinations might be less of a necessity compared to travelling to work, which allows people to be more influenced by factors such as perceived benefits and barriers.

A higher self-efficacy towards AT was related to being more likely to walk and cycle in all working young adults. This reinforces existing evidence on the importance of self-efficacy as a determinant of AT [27, 38, 48, 64] and physical activity [65] among adults in general. Also in all working young adults, perceived benefits and barriers towards AT were respectively positively and negatively related to the amount and the likelihood of cycling, which is in line with previous research in adults [27, 66]. In non-college educated participants only, perceiving more barriers towards passive transport was related to less minutes passive transport. Also in non-college educated participants, perceiving more benefits and less barriers towards AT was related to more minutes walking. However, in a study among lower and higher educated women, psychosocial factors (e.g. perceived barriers) did not influence the association between educational level and walking for transport [64]. Further research is required to investigate the link between attitude and walking/passive transport in non-college educated young adults in more detail. In all working young adults, social support, social norm and modeling towards AT and public transport were positively related to active and public transport. Previous studies also showed positive associations of social aspects with AT in (young) adults [27, 42, 48] and with public transport in older adolescents and adults [48, 67]. Modeling and social norm towards passive transport were also positively associated with passive transport. Especially among employed people, image and social status (e.g. presented to co-workers) have shown to be an important motive for driving a car [68, 69]. It is important to improve the image of AT and public transport and to create a positive social climate towards these transport modes in order to increase AT and decrease passive transport among working young adults.

Regarding environmental correlates, we found that for trips to other destinations, a higher land use mix diversity was associated with being more likely to walk, but less likely to cycle. A review on correlates of walking showed indeed a positive relation with land use mix diversity [70], but it has also been found that high walkable environments (with a high land use mix diversity) do not support cycling [71–73]. It might be that in areas with various destinations, distances are very short and pedestrians are omnipresent, hindering young people’s preferred cycling behaviors and speeds [42, 71]. Thus, appropriate cycling facilities are necessary. Next, living closer to work was positively related to cycling (in all participants) and negatively related to public and passive transport (only in college educated participants). Indeed, distance is one of the most consistent predictors of AT in all age groups [74–77]. All working young adults were more likely to commute passively when perceiving a lower density and a lower land use mix access, implying that areas with a high walkability (dense, good access to services) supported less car-dependent living [73]. Overall, these results are in line with previous reviews on environmental correlates in adults and youth [38, 76, 77], indicating that walkability of neighborhoods is important for AT (for walking and for less passive transport). However, appropriate cycling facilities are needed, especially in high walkable neighborhoods (e.g. separate cycling lanes and routes [77]) and at work (e.g. bicycle storage, showers).

In non-college educated working young adults, feeling safe from traffic and from crime was particularly important for their active and public transport use. They were more likely to cycle and less likely to use passive transport to all destinations when feeling safe from traffic. A previous review showed inconsistent associations between neighborhood traffic safety (both perceived and objective) and AT in adults in general [38]. However, traffic safety might be especially important for lower educated people as previous studies found social inequalities in traffic accidents [78–80]. Population groups who are disadvantaged in terms of income, education or quality of their residential areas are disadvantaged as users of the road transport system by sustaining injury more often than more advantaged population groups [79]. Furthermore, traffic accidents occur significantly more in neighborhoods that are poorer or more deprived [80]. Increasing traffic safety could help in promoting AT in non-college educated working young adults. Furthermore, non-college educated participants feeling safe from crime reported longer public transport trips to work and were more likely to use it to other destinations. Previous studies in adults found that perceptions of high crime [81] and an unsafe built environment related to personal safety (e.g. low visibility, low aesthetic quality) [82] were negatively associated to public transport use. Moreover, an inverse relationship between educational attainment and fear from crime has been found in existing research [83]. Decreasing neighborhood crime might increase public transport, but also decrease passive transport, as results showed that feeling safe from crime was related to being less likely to commute passively among all participants.

Some differences in transport to work between college and non-college educated participants might be more attributable to different occupational types, rather than to educational attainment. For example, college educated working young adults might be more likely to cycle to work because their occupations might be more centrally located within a city or business district, with reduced availability of low-cost car parking [84, 85] and no requirements of transporting heavy tools or specialized equipment. In addition, the importance of perceived safety (traffic and crime) for active and public transport use among non-college educated participants might be partly due to shift or night work with early mornings and late evenings during which darkness decreases perceived safety [86]. However, Bopp et al [87] found that occupation type did not influence transport mode choice to work.

Study limitations include the cross-sectional study design, so no causal relationships could be drawn from this study. Next, a self-reported questionnaire was used which could lead to participants’ over- or underestimating the use of questioned transport modes and distance to work. Since Belgium, and specifically Flanders, has good geographical and climatological conditions for cycling and a real ‘cycling culture’, caution should be taken when extrapolating the results outside Flanders [88]. Finally, results of the negative binomial model for walking, cycling and public transport to work in non-college educated working young adults need to be interpreted with caution, as post-hoc power analyses showed that statistical power was lower than 0.80. In future studies, a larger study sample would be needed to overcome this problem and to draw firm conclusions.

Strengths of this study include the chosen target group since evidence on transport behavior and its correlates is very limited in working young adults, especially among those with lower educational attainment. Next, walking, cycling, public transport use and passive transport use were questioned and analyzed separately but are all part of the same study, which allows to see broader patterns. Most previous studies solely focused on only one of these transport modes or combined them as active and passive transport. Following that, correlates of the different transport modes were investigated for both transport to work and transport to other destinations. Finally, psychosocial as well as environmental variables were investigated simultaneously.

## Conclusions

To encourage healthy transport behaviors among the important risk group of working young adults (both college and non-college educated), future interventions should focus on high self-efficacy, high perceived benefits and low perceived barriers towards AT (especially to destinations other than work), and on creating a positive social climate towards AT and public transport. Additionally, high walkable neighborhoods and good cycling facilities are important environmental factors for all working young adults. Improving walkability and cycling facilities might increase healthy transport behaviors among the whole population, as these factors have shown to be also important in youth and adults. Furthermore, the focus should be on increasing active and public transport participation among non-college educated working young adults and on encouraging more minutes of AT in college educated working young adults. Among non-college educated working young adults, perceived safety (traffic and crime) was important for active and public transport use. Increasing safety in their neighborhoods could help to increase healthy transport behaviors in this group. In college and non-college educated working young adults, both psychosocial and environmental variables were related to the four transport modes, but psychosocial variables were more important for transport to other destinations than to work. For transport to work, environmental variables, especially those related to the workplace, were more important.

## Supporting information

S1 Dataset Data obtained from the online questionnaire. (XLSX)
